# Osteopontin Expression and Its Role in Endometrial Cancer: A Systematic Review

**DOI:** 10.3390/cancers17132245

**Published:** 2025-07-04

**Authors:** Carmen Imma Aquino, Sakthipriyan Venkatesan, Arianna Ligori, Raffaele Tinelli, Elena Grossini, Daniela Surico

**Affiliations:** 1 Gynecology and Obstetrics Unit, Department of Translational Medicine, Università del Piemonte Orientale, 28100 Novara, Italy; 20027942@studenti.uniupo.it (A.L.); daniela.surico@med.uniupo.it (D.S.); 2 Laboratory of Physiology, Department of Translational Medicine, Università del Piemonte Orientale, 28100 Novara, Italy; sakthipriyan.venkatesan@uniupo.it (S.V.); elena.grossini@med.uniupo.it (E.G.); 3 Department of Obstetrics and Gynecology, “Valle D’Itria” Hospital, Martina Franca, 74015 Taranto, Italy; raffaele.tinelli@asl.taranto.it

**Keywords:** osteopontin, tumors, endometrial cancer, tumorigenesis, systematic review

## Abstract

Background/Objectives: Osteopontin (OPN) is a 34 kDa protein that is extensively phosphorylated and rich in aspartic acid, produced by a single-copy gene, and altered by post-translational processes. In several diseases, OPN has been discovered to play a direct role in immunological and inflammatory responses. It is also important in kidney stone disease, preeclampsia, cardiovascular disease, endometriosis, and cancer, among other pathological conditions. It is a crucial extracellular matrix molecule involved in oncology, due to its ability to bridge the gap between inflammation and carcinogenesis. Methods: Our systematic review has as PICO “Does Osteopontin have possible etiological and prognostic correlations in patients affected by endometrial carcinoma?” Based on online data collected from PubMed, Scholar, Embase, Scopus, and other sources, a preliminary analysis was conducted. A keyword search for “Osteopontin” AND “tumors”, “endometrial cancer”, and other related terms was used to identify the publications. The relevance of scientific research was used to select articles in English. Results: For our systematic review, the citation search yielded nine articles on the topic. At the endometrial level, OPN plays a role in a variety of biological processes, including angiogenesis, metastasis, altered tissue remodeling, immunological responses, cell adhesion, and migration. Conclusions: With established direct correlations and a potential role in the assessment of the diagnosis and prognosis of the disease, OPN participates in endometrial cancer, drawing more and more attention from researchers.

## Simple Summary

Osteopontin (OPN) is an arginine–glycine–aspartic acid glycoprotein, a component of the extracellular matrix that can bind to integrin receptors at the cell surface to mediate cell adhesion and migration. The human gene contains seven exons and maps to chromosome 4 (4q13). OPN has been recognized as a pleiotropic cytokine considered in a wide array of biological processes. It is a multifaceted protein that is involved in the pathogenesis of several diseases, including cancer. It is responsible for various activities, some related to cell viability and the secretion of interleukins, interferon-γ, integrin αvB3, nuclear factor-kappa B, with action on osteoclasts and inflammation through macrophages and T cells. Although OPN has been assigned many roles in oncological processes of invasion, metastasis, and angiogenesis, little is known about its potential involvement in endometrial cancer.

## 1. Introduction

The “*Secreted Phosphoprotein 1*” *(SPP1)* gene encodes the 44–70 kDa protein known as Osteopontin (OPN). This human gene corresponds to chromosome 4 (4q13) and has seven exons. It is a highly phosphorylated glycoprotein that contributes to cell signaling pathways by interacting with integrins (e.g., αvβ3 and αvβ5) and cluster of differentiation 44 (CD44), among other cell surface receptors [1,2]. Although OPN was first discovered through a variety of methods, its role in mineralization, bone homeostasis, and remodeling was the focus of early studies. It has been found that OPN has an intracellular domain that can facilitate different biological functions of the molecule. OPN was found to be directly involved in inflammatory and immune responses in several pathologies. Kidney stone disease, preeclampsia, cardiovascular disease, endometriosis, and cancer are among the pathological situations in which OPN also plays an important role [1,2]. The stromal uterine cells of the decidua and the secretory epithelial cells of the endometrium both exhibit significant levels of OPN expression [3]. The OPN messenger ribonucleic acid (mRNA) has also been shown as being selectively localized in endometrial epithelial cells, according to the in situ hybridization investigations. OPN immunostaining has been observed in the apical regions of the surface (luminal) epithelium and glandular secretions. The pathophysiology of endometriosis, a benign condition with certain characteristics similar to malignant tumors, may also be influenced by OPN isoforms, with overexpression in both endometriosis and adenomyosis [4,5], compared to the normal endometrium and through the activation of the phosphoinositide 3-kinases (PI3K) and nuclear factor kappa-light-chain-enhancer of activated B-cell (NF-ĸB) pathways. Furthermore, the expression of OPN could be influenced by hormones such as progesterone, while the β3 integrin subunit is upregulated by the epidermal growth factor or heparin-binding epidermal growth factor.

OPN has a significant role in various physiological and pathological patterns, including cell adhesion, migration, and immune regulation, acting on cellular viability and the secretion of interleukins, interferon-γ, integrin αvB3, and NF-ĸB, with action on osteoclasts and on inflammation through macrophages and T cells [6,7]. This protein is regulated by various substances such as cytokines and growth factors, and is crucial for modulating gene transcription, translation, and post-translational modifications, thereby influencing a wide range of cellular activities [7].

Due to its ability to intersect inflammation and tumorigenesis, OPN is described as a key extracellular matrix molecule implicated in the oncology of different parts of the body [6,7]. In fact, OPN contributes significantly to the occurrence and progression of several cancers. Through the activation of the PI3K-AKT-GSK/3b-b/catenin and MAPK (mitogen-activated protein kinase) signaling pathways, OPN promotes macrophages to retain the M2 phenotype in colorectal cancer. By binding to integrin and CD44 receptors and activating WNK-1 (lysine deficient protein kinase 1), PRAS40 (proline-rich AKT substrate 40), and other signaling pathways, OPN can increase angiogenesis in breast and in liver cancer by triggering the αvβ3-NF-κB signaling pathway. FAK (Focal Adhesion Kinase)/AKT (Protein Kinase B), ERK (Extracellular Signal-Regulated Kinase), RON (receptor tyrosine kinase), MAPK signaling pathways, VEGF (Vascular Endothelial Growth Factor), COL11A1 (Collagen Type XI Alpha 1), and other angiogenic factors are substantially expressed in non-small-cell lung cancer [8].

Through binding with integrin receptors or CD44 receptors, OPN acts as a complex intracellular “signaling traffic network” (i.e., key kinases, signaling pathways, and transcription factors) [9]. OPN overexpression is closely associated with the development of cancer progression, including proliferation, metastasis, angiogenesis, apoptosis resistance, drug resistance, and immunosuppression, and may also be an independent prognostic biomarker for a variety of cancers [10,11]. It has been implicated in several tumors, such as head, neck, lungs, breast, liver, stomach, colon, and endometrial cancer [12]. Multiple functions in oncological processes have been attributed to OPN, but its possible role has been poorly understood for endometrial cancer (EC) [13,14]. EC is one of the most common gynecological cancers in developed countries, with a constantly increasing incidence (currently approximately 417,000 new cases per year), it is rising in tandem with the global epidemic of obesity [15]. Environmental risk factors are linked in several ways to this oncogenic pathway, potentially based on chronic inflammation and related to oxidative stress, DNA (deoxyribonucleic acid) methylation, telomere length, and immune function. Early diagnosis is the key to improving survival, which at 5 years is less than 20% in advanced disease and is over 90% in early-stage disease [16,17,18,19].

The role of OPN in EC is complex, with evidence suggesting its involvement in tumor progression, metastasis, and prognosis [14]. As demonstrated in vitro and in vivo studies, OPN has been expressed in EC-affected cells, even resulting in overexpression; decreased expression showed reduced migration and invasive capacity [20,21]. OPN also appears to be involved in apoptotic processes [3], and the inhibition of OPN expression in endometrial lines can significantly reduce cell migration and tumor angiogenesis [22].

In addition, OPN has become a potentially useful biomarker for cancer diagnosis and treatment. There are currently few biological indicators for the early detection of EC that have been validated. Recent developments in machine learning and high-throughput technologies present novel and exciting avenues for biomarker discovery, particularly when combining imaging and genomic science [23]. Understanding the molecular mechanisms and pharmacological effects of OPN in cancer development could lead to new targets for improving cancer diagnosis and treatment [24].

### Aims

How does the available data correlate with OPN, and how is it possible to describe the integration of prognostic and predictive tools, and their application in clinical practice for patients with endometrial cancer?

This review aims to explain how OPN could be associated with EC, from an etiological and therapeutic point of view, to provide deep information on the topic, to personalize the approach, and to possibly improve patients’ chances of a better therapeutic response and quality of life. In fact, the purpose of this research is to review the literature and ascertain how this information should be interpreted to provide future clinical practice recommendations.

## 2. Materials and Methods

### 2.1. Identifying Target Population

The studied sample consists of tissues from cases affected by endometrial cancer.

### 2.2. Systematic Literature Search

**Figure 1:**
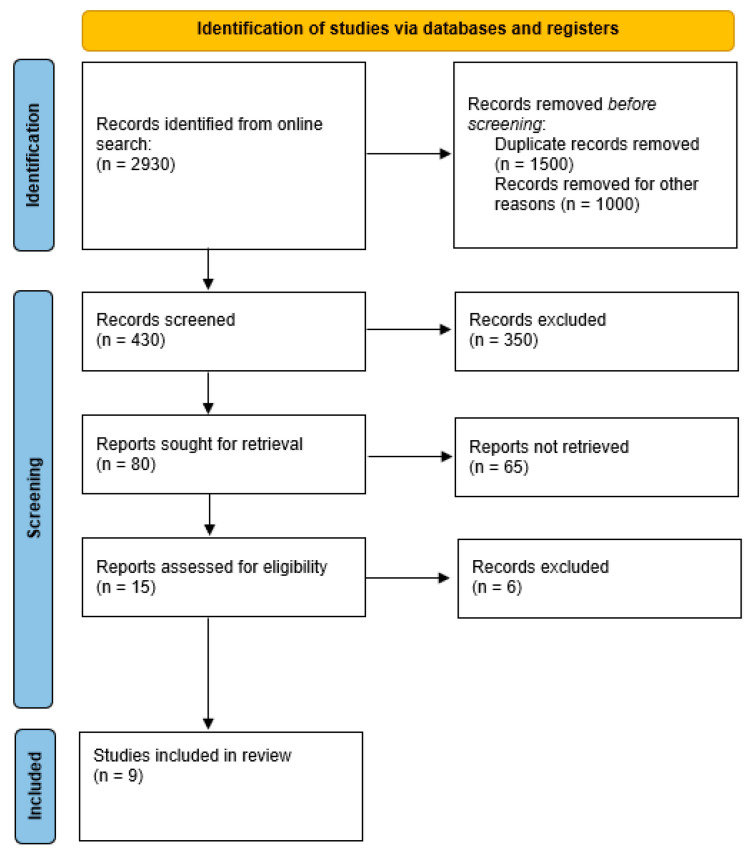
PRISMA flow chart.

We elaborated a systematic review based on this PICO, “Does Osteopontin have possible etiological and prognostic correlations in patients affected by endometrial carcinoma?” A search of scientific literature was undertaken for the period from January 2005 to June 2025. The preliminary analysis was carried out based on in vitro and in vivo data gathered from PubMed, Scholar, Embase, Scopus, etc. The inclusion criteria were the identification of the articles based on a keyword search for “Osteopontin” AND “tumors”, “endometrial cancer”; in the English language, filtered according to the relevance of the scientific research. We excluded research written in languages other than English and that was related to other molecules and cancers. Two authors double-checked the appropriateness of the literature search. The authors have reviewed and edited the output and take full responsibility for the content of this publication. This study was conducted by following the Preferred Reporting Items for Systematic Reviews and Meta-Analyses (PRISMA). This study has been registered in the International Prospective Register of Systematic Reviews (PROSPERO; registration number CRD420251078792, link in Supplementary Materials: https://www.crd.york.ac.uk/PROSPERO/view/CRD420251078792 accessed on 23 June 2025). Citation searching provided nine articles on the topic for our systematic review (PRISMA flow chart, Figure 1).

## 3. Results

In Table 1, we analyzed in detail articles about OPN and EC, considering the possible correlations since OPN expression is upregulated in various malignancies. Concomitant changes were seen in the expression of OPN binding to cell surface receptors, cell cycle regulation genes, cell invasion, and colony formation of the tumor cells. The inhibition of anchorage-independent growth was described in the presence of metabolic inhibitors of the PI3K, Src (Proto-oncogene tyrosine-protein kinase), and integrin signaling patterns. Nine articles described evaluations in vivo and in vitro regarding EC and OPN [13,14,20,21,22,25,26,27,28]. In most of the articles, OPN seems to be involved in tumor promotion and/or metastasis in human EC (Table 2). In a minority of publications, the correlation seems to be different [26].

Studies suggest several key roles for OPN in the context of EC, including for therapy and prognosis (Table 1). The available data suggest the following key roles for OPN in the context of EC: tumor progression and aggressiveness, immune modulation, angiogenesis, metastasis, and invasion.

### 3.1. The Functional Role of Osteopontin in EC

#### 3.1.1. OPN in Tumor Progression and Aggressiveness

OPN is involved in tumor promotion in human EC, maybe through increased expression. Elevated OPN levels have been found in the samples of patients with EC compared to healthy controls. It is believed to contribute to tumor progression, as the expression correlates with higher tumor grades and advanced stages of cancer [8,27].

A notable reduction in cell viability and an increase in apoptotic cell death have been seen in EC cells when OPN gene expression is reduced. The status of OPN in the THESC, RL95, Hec1A, and Ishikawa cell lines has been examined. Hec1A and Ishikawa cells’ OPN mRNA and protein expression levels have been assessed following OPN–small interfering RNA (siRNA) transfection. The ability of EC cell lines (Hec1A and Ishikawa cells) to proliferate is altered when OPN gene expression is reduced [11,25,29].

#### 3.1.2. OPN and Immune Modulation

OPN promotes Epithelial–Mesenchymal Transition (EMT), a process by which epithelial cells lose their characteristics and gain migratory and invasive properties. This contributes to cancer cell metastasis and resistance to therapies [12,25]. OPN is expressed by many immune cells (e.g., T cells, B cells, natural killer cells, and macrophages). The expression of OPN binding to cell surface receptors, which regulate the cell cycle and the tumoral propensity for colony formation and cell invasion, has been shown to change concurrently. When recombinant OPN is present, the decreased colonization potential that had occurred in the absence of OPN is reversed. Metabolic inhibitors of the PI3K, Src, and integrin signaling pathways have been shown to inhibit anchorage-independent growth; this effect is mitigated by exogenously administered OPN [30,31,32]. These results may describe the involvement of OPN in EC, especially its cancer-promoting properties, which could open the door to novel methods of treating EC clinically. The main actions studied in solid tumors are related to:

- Immune Evasion: OPN has been shown to interact with immune cells, promoting tumor immune evasion. It can affect the function of T cells, macrophages, and natural killer (NK) cells, thus enabling the tumor to escape immune surveillance.
- Inflammation and Tumor Microenvironment: OPN contributes to the inflammatory tumor microenvironment by recruiting inflammatory cells and promoting the secretion of pro-inflammatory cytokines, which can enhance tumor growth and resistance to chemotherapy. It has been demonstrated that poor prognoses and survival rates in a variety of human cancers are linked to the expression of OPN as a tumor microenvironment component in cancer tissues, plasma, and serum. According to recent research, OPN uses a variety of mechanisms to promote tumor growth and aggressiveness [6].

In Kariya et al.’s review, it is reported that OPN promotes tumor progression, such as tumor growth, invasion, angiogenesis, and immune modulation, as well as metastasis and chemoresistance by suppressing the immune system [10]. The modulation is enacted through interaction with integrins and CD44 receptors, but also by post-translational modification, such as proteolytic processing by proteases, phosphorylation, and glycosylation. To influence the complete immune response, OPN’s primary purpose during inflammation is to activate various leucocytes, triggering a functional response and causing cytokine release. OPN mainly promotes macrophage movement, accumulation, and retention at injury sites. It can also enhance Th1 cell-mediated immunity and encourage monocyte differentiation. In fact, monocyte adhesion, migration, differentiation, and phagocytosis are among the immune cell processes that OPN regulates. OPN increases NK cell migration and activation; it also affects dendritic cells and neutrophil recruitment. By promoting Th1 and Th17 differentiation and suppressing Th2 cytokine production, OPN contributes to Th cell polarization [33,34,35,36]. In EC, elevated OPN expression has been connected to rising invasion and metastasis. According to in vitro research, OPN contributes to EC cell migration through the modulation of ECM.

#### 3.1.3. Angiogenic Role of OPN in EC

OPN is overexpressed in cancer-associated endometrial samples from in vivo and in vitro experiments [9,19]. OPN stimulates angiogenesis by promoting endothelial cell migration and tube formation, which are necessary for tumor growth and metastasis. It also increases the expression of VEGF in EC cells [9]. Since OPN is related to abnormal angiogenesis in EC, it could be a future target of therapy. Moreover, a favorable microenvironment is necessary for the growth and evolution of cancers, in which tumor cells recruit bone marrow accessory cells to start angiogenesis, and impact normal resident cells like fibroblasts and endothelial cells. Both stromal and tumor cells form the extracellular matrix (ECM), which is made up of both structural and functional proteins. Tumors cause stromal alterations, such as the recruitment of mesenchymal stem cells, leucocytes, and endothelial cells. Local fibroblasts are also reprogrammed to become cancer-associated. These steps are necessary to promote abnormal angiogenesis [21]. As reported in the next paragraph, OPN is involved in ECM dynamics and in this way could also affect angiogenesis.

#### 3.1.4. Metastasis and Invasion

**Figure 2:**
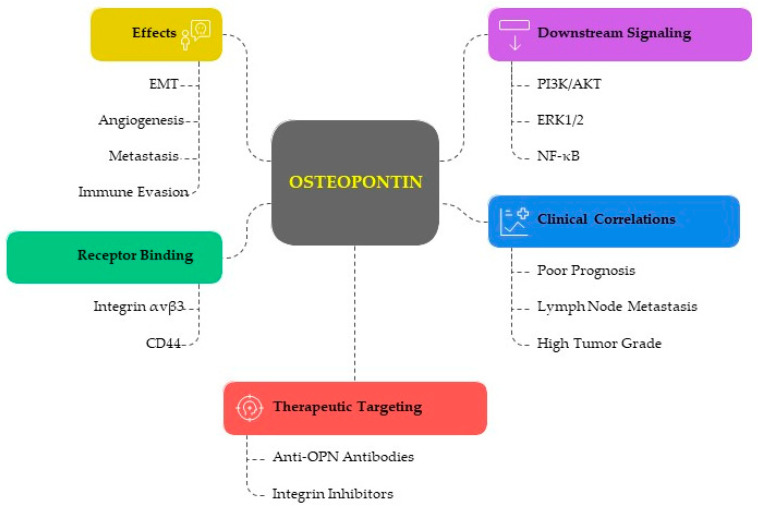
OPN in EC Progression. CD: cluster of Differentiation; EMT: Epithelial–Mesenchymal Transition; ERK1/2: Extracellular Signal-Regulated Kinases 1 and 2; NF-κB: Nuclear Factor Kappa-Light-Chain-Enhancer of Activated B cells; OPN: Osteopontin; PI3K/AKT: Phosphoinositide 3-kinase/Protein Kinase B. Created through BioRender.com (accessed on 3 June 2025).

Metalloproteinases (MMPs) are enzymes that break down the ECM, promoting cancer cell invasion and metastasis. OPN acts on cell proliferation, migration, and invasion, also influencing EMT-related factors and MMP-2 expression, through protein kinase B (PKB/AKT) and extracellular regulated protein kinase (ERK1/2) signaling pathways [12]. Being an essential part of the cellular microenvironment, ECM is crucial for the growth and maintenance of healthy tissues, as well as homeostasis. Numerous processes, including endometriosis, infertility, cancer, and metastasis, are influenced by the abnormal dynamics of ECM. In cancer, MMPs have a significant effect on tumor growth. MMPs are essential for the breakdown of the basal membrane and ECM components communicating with a variety of particular substrates, including collagen, elastin, fibronectin, aggrecan, fibulin, laminin, tenascin, vitronectin, versican, and nidogen. It was discovered that EC had elevated levels of MMPs, specifically MMP-1, MMP-2, MMP-9, and MMP-11. Increased concentrations of MMP-2 and MMP-9 have also been related to the FIGO (International Federation of Gynecology and Obstetrics) stage and are associated with poor prognosis in EC. Although their function is as yet unknown, EC also seems to be linked to elevated levels of disintegrin and MMPs with thrombospondin motifs [37]. MMPs cleave OPN, increasing its activity. Other variables could influence these oncological patterns. For example, obesity in post-menopause increases the incidence of EC and the death of EC patients via a mechanism frequently linked to estrogens. Therefore, obesity influences OPN expression in EC, which may promote the synthesis of estradiol and, in turn, the association between obesity and estrogen-dependent cancers. OPN and MMPs are both elevated in obesity, and MMP-cleaving OPN is capable of inducing aromatase activity in human adipocytes. OPN increases the expression of PI3K and ERK1/2, and migration and proliferation in cell cultures [25,28,38]. The estrogen-synthesizing enzyme aromatase has been shown to be induced in preadipocytes by tumor necrosis factor alpha (TNFα) and interleukin 1; however, the effect of OPN on aromatase expression in adipose tissue is still unknown. In vitro, in adipocytes, OPN is able to boost aromatase mRNA dramatically. In visceral and subcutaneous adipose tissue, aromatase expression has been strongly correlated with the gene expression of OPN and other MMPs [38]. Figure 2 illustrates the multifaced role of OPN in the progression of EC. Table 2 describes the mechanisms involved in the effects of OPN in EC and its potential as a diagnostic, prognostic, and therapeutic tool.

### 3.2. Clinical Implications and Prognostic Value

To improve the staging and to evaluate possible treatments, new indicators of progression for EC are required [39]. Metabolic inhibitors of the PI3K, Src, and integrin signaling cascades have been shown to decrease anchorage-independent growth. OPN has clinical potential in EC: targeting tumor OPN molecules may help EC patients by resetting gene networks that are relevant to metastases.

#### 3.2.1. Diagnostic Marker and Serum and Tissue Biomarker

It remains unclear how OPN could function as a biomarker for EC. OPN may be used for diagnosis due to the high amounts of the protein found in EC patients’ serum and tissue samples. According to Zou et al., in fact, serum OPN levels may be used as a non-invasive biomarker for EC detection and illness progression tracking [40]. Women affected by cancer had significantly higher plasma OPN levels than healthy controls (*p* < 0.001). OPN has also been used as an independent predictor of disease-free survival in FIGO stage 1 EC, correctly identifying 18 of 29 cases (62.1%) that CA125 failed to detect (*p* = 0.035) [13,25].

OPN is marginally elevated in benign endometrial hyperplasia, whereas it is roughly three to five times higher in extremely malignant endometrial cancer tissue. There are significant differences in the level of OPN expressed between oncological grades 1 and 3 or higher (*p* = 0.001) [41]. Kaplan–Meier survival analysis, however, reveals that there is no significant correlation between the patients’ shorter survival times and higher levels of OPN expression. OPN in ovarian endometrioid carcinoma (OEC) and endometrioid endometrial cancer (EEC) was compared in another investigation: 63 cancer cases in all (33 EEC and 30 OEC) [28]. OPN expression was detected in 15 (50.0%) of the 30 EECs and 14 (50.0%) of the 28 OECs. The percentage of positive cytoplasmic OPN staining in the EECs and OECs did not differ significantly (12.8 vs. 10.4; *p* = 0.6811). In both the EECs and OECs, there was a substantial link between relative mRNA and protein expression levels, but not between relative DNA and mRNA levels. The OPN expression of the EECs and OECs did not differ significantly [28].

New tumor markers and indicators of tumor progression are needed for improved staging and for the better assessment of treatment in many cancers, including EC; the therapeutic targeting of tumor OPN molecules could result in clinical benefit.

#### 3.2.2. Prognostic Significance and Therapeutic Targeting

In patients with EC, high OPN concentrations seem to be linked to poor prognoses. Poor survival outcomes are indicated according to tumor size, depth of invasion, and lymph node metastases, all of which are correlated with elevated OPN levels [20]. The possibility of a targeted therapy relying on OPN is being investigated because of the crucial role in immune evasion, metastasis, and EC development. Tumor growth and metastasis may be prevented by targeting OPN’s interactions with integrins, CD44, or through downstream signaling pathways [4]. Furthermore, OPN is regarded as a separate predictor of both overall survival and progression-free survival in EC. Increased OPN levels may be related to the malignant transformation of EC cells. In contrast to normal endometrial tissue, EC samples show higher levels of OPN expression: OPN was marginally elevated in benign endometrial hyperplasia [42].

The role of OPN as a biomarker in EC has not been definitively established [13]. Lower OPN values have been significantly associated with FIGO stage, lymph node metastasis, and the depth of myometrial invasion (*p* < 0.01), with a significant difference in overall survival (OS) and disease-free survival (DFS) (*p* < 0.001). Moreover, OPN expression is an independent predictive factor for OS (*p* = 0.043) and DFS (*p* = 0.027) in EC patients. Spearman’s rank correlation analysis has described a positive correlation between serum OPN levels and adiponectin (r = 0.455, *p* < 0.001), and a negative correlation between serum OPN levels and leptin (r = −0.307, *p* < 0.001) [24].

#### 3.2.3. Involvement of OPN in Treatment Resistance

OPN, in its multiple functions, also seems to influence the therapeutic aspect, impacting the efficacy of radiotherapy [41]. This is one of the most effective therapies for some stages of EC, which has also been shown to reduce the likelihood of local recurrences following surgery, but a significant portion of patients have tumor recurrence as a result of radioresistance mechanisms. Numerous fundamental cellular signaling pathways and processes, including the immune system, growth factor receptor signaling, DNA damage repair mechanisms, and the PI3K/AKT, MAPK, and NF-κB pathways, are known to influence responses to radiation in EC [19,41]. Figure 2 illustrates the multifaced role of OPN in the progression of EC. In Table 2, we described the mechanisms involved in the effects of OPN in EC and its potential as diagnostic, prognostic and therapeutic tool are shown.

**Table 2:** Role of OPN in EC.

Category	Key Findings	Mechanisms/Pathways	Clinical Implications	Citations
Tumor Progression & Aggressiveness	Overexpressed in EC tissues in serum vs. healthy controls, Correlations with advanced tumor grade/stage OPN knockdown reduces proliferation, increases apoptosis	PI3K/AKT, cell cycle regulation	Tumor marker, Therapeutic target potential	[8,11,25,27,29]
Immune Modulation	Promotion of EMT and immune escape, Regulation of T cells, NK cells, macrophages, DCs, Enhancement Th1/Th17, suppression of Th2 responses	Integrins, CD44 interactions post-translational modifications (phosphorylation, glycosylation)	Immune-based therapy target	[9,12,25,30,31,32,33,34,35,36]
Tumor Microenvironment	Promotion of inflammation, cytokine secretion Recruitment of immune/ inflammatory cells	NF-κB, integrin/PI3K/Src signaling pathways	Targeting OPN may disrupt tumor-supporting microenvironment	[6,30,31,32]
Angiogenesis	Stimulation of endothelial migration and tube formation, Increase in VEGF expression	OPN-VEGF axis ECM remodeling and angiogenic signaling	Anti-angiogenic therapy potential	[9,19,21]
Metastasis and Invasion	Enhancement of EMT, migration, invasion, Upregulation of MMP (particularly MMPs-2/9)	PI3K/AKT, ERK1/2 MMP-mediated ECM degradation	Linked to aggressive EC phenotype	[12,25,28,37,38]
Obesity & Hormonal Link	OPN is upregulated in obesity, OPN increases estradiol synthesis	OPN-MMP-aromatase axis TNFα, IL-1	Explains obesity-related risk of estrogen-dependent EC	[25,28,38]
Prognostic Value	Higher OPN linked to poor prognostic factors (grade, invasion, lymph node metastasis) Opposite findings on survival correlation	Correlation with adiponectin/leptin	Prognostic marker (in specific subtypes/stages)	[13,24,25,41]
Diagnostic Utility	Higher serum/tissue OPN in EC vs. controls. It may outperform CA125 in early-stage EC detection		Non-invasive diagnostic biomarker	[13,25,41,42]
Therapy Resistance	Contributes to radiotherapy resistance	PI3K/AKT, MAPK, NF-κB DNA damage repair involvement	Blocking OPN could sensitize EC cells to radiotherapy	[19,43]
Therapeutic Target	OPN inhibitors may block proliferation, invasion, immune evasion, Resetting gene networks linked to metastasis	Targeting integrins, CD44, ECM, downstream Signaling	Candidate for personalized therapy or combination regimens	[4,9,40]

AKT: protein kinase B, CA125: cancer antigen 125, CD44: cluster of differentiation 44, DCs: dendritic cells, EC: endometrial cancer, ECM: extracellular matrix, EMT: epithelial–mesenchymal transition, ERK1/2: extracellular signal-regulated kinases 1 and 2, IL-1: interleukin-1, MAPK: mitogen-activated protein kinase, MMPs: matrix metalloproteinases, NK cells: natural killer cells, OPN: osteopontin, PI3K: phosphatidylinositol 3-kinase, TNFα: tumor necrosis factor alpha, VEGF: vascular endothelial growth factor.

## 4. Discussion

OPN is a small integrin-binding ligand N-linked glycoprotein first identified in 1986 in osteoblasts. Specifically, OPN has been demonstrated to interact with integrins and has several roles associated with various organs, including the uterus and placenta during the estrous cycle and pregnancy [44]. Although OPN has been examined alongside other markers, a thorough analysis of the published findings and EC is still missing. The idea that marker combinations may be more effective than single molecules in terms of diagnosis or prognosis has gained traction in recent years. The combination of OPN with other cancer-specific markers, synergizing different biomolecules (i.e., fibrinolytic system, calcium homeostatic proteins, squamous cell carcinoma antigen, NF-κB pathways, proteases, etc.), and functionally convergent elements (i.e., angiogenesis, motility/adhesion, extracellular matrix, bone, etc.) have been studied [39]. Moreover, OPN can mediate the development of tumors of several types, such as the cervical, ovarian, and endometrial cancers that are the most common feminine tumors. EC is a prevalent malignancy in Western nations nowadays. If detected early, EC has a good prognosis and is limited to the uterus in 75% of cases. However, as the disease worsens, the prognosis dramatically deteriorates [14].

Multiple classifications are based on histopathological traits (e.g., endometrioid, serous, etc.) or clinical and endocrine parameters (e.g., types 1 and 2). The biological, clinical, and molecular characteristics of these divisions exhibit significant heterogeneity. In order to guide the therapy, the current FIGO evaluation gives prognostic information, designating grade 1 and 2 tumors as “low grade” and grade 3 tumors as “high grade.” In addition, there are four genomic subtypes: polymerase E mutant, mismatch repair deficient, copy number low/p53 wild-type, and copy number high/p53 aberrant. Since each oncological variety exhibits unique molecular aberrations that permit additional subclassifications, this additional correlation could be essential [44].

OPN in EC has been shown to have pleiotropic effects; to date, conflicting data are also present in the literature. Al Maghrabi et al. controversially discovered that there was no correlation between myometrial invasion, lymph vascular invasion, surgical resection margin, tumor size, histological type, FIGO tumor grade, or lymph node metastasis. Eighty percent of the proliferative and secretory phase samples from the normal human endometrium showed strong OPN expression with a consistent cytoplasmic location in epithelial glandular cells. Additionally, OPN immunostaining was higher in 100% of non-tumoral tissues compared to 64.8% of oncological samples (*p* < 0.001) in the non-neoplastic endometrium, and it was linked to better overall survival [26].

Comparing the DNA, RNA, and protein levels of OPN in EEC and OEC, there was no significant difference in positive cytoplasmic OPN staining [28].

Most of the research to date correlates OPN with EC, and OPN might play a different role in the pathogenesis and diagnosis of EC [27]. The identification of cancer, the evaluation of its progression and its prognosis/reaction to treatment have been clinical parameters of interest. Cancer antigen 125 (CA125), B, alpha-fetoprotein, inhibin, carcinoembryonic antigen (CEA), squamous cell carcinoma antigen, carbohydrate antigen 19-9 (CA19-9), cancer antigen 27-29, human epididymis protein 4 (HE4), transthyretin, immunosuppressive acidic protein, leptin, cancer antigen 15-3 (CA15-3), cytokeratin 19, and thymidine kinase are also frequently studied as biomarkers for gynecological cancers. For these capacities, certain marker combinations with OPN could be promising. Those pertinent biomarkers have been found to have substantial *p*-values and positive predictive values for EC. The combined tumor markers CA19-9, CA125, leptin, thymidine kinase, CEA, CA15-3, and HE4 have been shown to have a 95% sensitivity and a 96% specificity for detecting EC. The positive predictive value of combining CA19-9, CA125 levels, HE4, CA15-3, leptin, thymidine kinase, and CEA for the detection of EC was 93%. There has been an increasing recognition that marker combinations may have better diagnostic or prognostic value than individual markers [45]. As evident in the literature, the combination of blood markers such as CA125 and HE4 could result in early diagnosis [27]. CA19-9 was also more effective in comparison to CA125, with the highest specificity being 95.7% and the highest sensitivity being 99.7% for CA125: the possible relationship with OPN could be highly interesting. To fully deepen the diagnostic and prognostic potential of OPN, more extensive research is required, given the current great interest in multiplex marker panels [39]. Preoperative screening may aid in customizing the prognosis and outcomes of each patient [46]. OPN in plasma examination properly identified 62.1% of FIGO stage 1 carcinomas, for which onco-markers such as CA125 had not changed significantly. Additionally, it was discovered that increased OPN values were an independent predictor of DFS (*p* = 0.035) [13]. OPN has been linked to these cancer-specific indicators. The clinical parameters of interest related to OPN are the identification of cancer, the assessment of progression, and prognosis/treatment response. Although OPN has not been thoroughly studied as a potential biomarker, its distinct expression in EC could identify, for example, the biological diversity of endometrioid and serous malignancies. Furthermore, to date, OPN has not been definitively assessed as an EC biomarker or studied in relation to the latest molecular classification [47]. OPN has yet to be investigated as a predictive factor, according to studies on transcriptomics conducted on tissue from EC patients [48].

To adequately complete the diagnostic and prognostic potential of OPN, more extensive research is required, given the present great interest in multiplex marker panels and the possible therapeutic influence. In fact, increased vulnerability to radiation therapy also seems to be linked to the inhibition of OPN expression [3].

In this review, we first provide an overview of how OPN promotes tumor progression by acting on tumor growth, invasion, angiogenesis, and immune modulation, as well as metastasis and chemoresistance. These results may open up a novel therapeutic strategy: OPN could have important roles in controlling the growth of EC cells and may suggest a novel target pathway for the treatment of EC

As a main limitation of our evaluations, the sample size of the included articles could be incremented in future research. Moreover, the quality and risk bias of articles were not accessible for every case, with possible heterogeneity in the evaluation of in vitro and in vivo *studies*. Another limiting aspect is the conflicting results on the prognosis of EC according to the OPN level.

## 5. Conclusions

EC is still a leading cause of death worldwide. The tumor microenvironment component and OPN levels in tissues, plasma, and serum have been associated with poor prognoses and survival rates in various human cancers. OPN may drive tumor development and aggressiveness through various strategies.

### Future Prospects

OPN plays a multifaceted role in the pathogenesis of EC, contributing to tumor progression, invasion, metastasis, and immune evasion. Elevated OPN expressions in EC tissues and serum could serve as potential diagnostic and prognostic biomarkers. Moreover, targeting OPN and its signaling pathways offers promising therapeutic opportunities. Further research, including clinical trials, is needed to fully elucidate the role of OPN in EC and to develop effective therapies targeting this pathway.

## Figures and Tables

**Table 1:** Role of OPN in EC: Literature Review.

Authors/Year	Country	Sample	Main Results	Mechanisms	Clinical Significance
Kardelen et al., 2024 [25]	Aydın Adnan Menderes University Biochemistry Laboratory, Turkey	In vitro— HUVEC and Ishikawa human Endometrial Adenocarcinoma cells	With increasing OPN levels, cells showed increased proliferation and migration. At 400 ng/mL, OPN induced EMT	Involvement of PI3K and ERK1/2 pathways	OPN may serve as a prognostic indicator for EC progression
Li et al., 2015 [13]	Dalian Medical University, China	In vitro— HEC-1A cell line	OPN promotes EC cell proliferation, migration, invasion, and EMT	Activation of AKT/ERK1/2 signaling and increased MMP-2 expression	Potential for OPN-targeted therapies in EC treatment
Hahne et al., 2013 [20]	University of Würzburg, Germany	In vitro— Endometrial cell lines Ishikawa, Hec-1A, and An-3CA as well as M14/MDA-MB435	Lower OPN expression reduced invasion/migration and increased sensitivity to radiation therapy. OPN increased apoptosis	Effects of invasion/ migration related to OPN level of expression. Reduction in PARP and caspase-3	Targeting OPN could enhance radiosensitivity in EC
Ramachandran et al., 2013 [21]	Keimyung University School of Medicine, South Korea	In vitro — Hec1A and Ishikawa cells	Reduced OPN expression altered proliferation; recombinant OPN restored colony formation	Effects are mediated via PI3K, Src, and integrin signaling pathways	Highlights potential for novel targeted therapies involving OPN pathways
Du et al., 2009 [22]	Shandong Cancer Hospital; China	In vitro and in vivo— ISK cells and mouse xenografts	Silencing OPN decreased migration (67.4%), invasion (51.2%), and tumor angiogenesis	Effects on angiogenesis caused by binding to αVβ3 integrin and enhancement tube formation and invasiveness	OPN with a pro-tumorigenic role; possible anti-angiogenic strategies targeting OPN
Al Maghrabi et al., 2020 [26]	Department of Pathology, King Abdulaziz University, Jeddah, Saudi Arabia	Human tissue samples—71 EC vs. 30 non-neoplastic endometrial tissues	100% of non-neoplastic tissues demonstrated high OPN immunostaining; 64.8% of EC cases showed an increase in OPN. Higher OPN seems to be linked to better overall survival. No association with clinicopathological features		OPN could serve as a diagnostic and prognostic biomarker in EC
Cho et al., 2009 [14]	Yonsei University College of Medicine, South Korea	Clinical study— 56 EC patients vs. 154 benign controls	Plasma OPN was significantly higher in EC patients; detecting early-stage disease missed by CA125	Elevated OPN is associated with immune/ inflammatory responses	OPN is an independent predictor of DFS and could complement CA125 in diagnosis
Briese et al., 2006 [27]	University Clinic Hamburg-Eppendorf, Germany	Tissue analysis—20 normal benign, 17 hyperplastic, 43 EC tissues	67.4% of EC showed strong OPN expression; serous carcinomas had highest OPN. Loss of OPN correlated with higher malignancy grade	Effects are related to the creation of functional complexes with CEACAM1; post-translational effects possible	OPN contributes to biological diversity between EC subtypes; may aid in subtyping and prognosis
Hashiguchi et al., 2006 [28]	Osaka City General Hospital, Japan	Comparative study— 30 ovarian OEC and 33 EEC	50% of both OEC and EEC showed OPN expression; no significant difference between groups. Strong correlation between OPN mRNA and protein levels	Gene expression patterns suggest transcriptional regulation of OPN	May help in understanding molecular similarities/differences between OEC and EEC

Akt: protein kinase B, CA125: cancer antigen 125, CEACAM1: Carcinoembryonic antigen-related cell adhesion molecule 1, DFS: disease-free survival, DNA: deoxyribonucleic acid. EC: endometrial carcinoma, EEC: endometrioid endometrial cancer, EMT: epithelial–mesenchymal transition, ERK: extracellular regulated protein kinases, FIGO: International Federation of Gynecology and Obstetrics, mRNA: messenger ribonucleic acid, MMP: matrix metalloproteases, OPN: osteopontin, OEC: endometrioid cancer, OS: overall survival, PARP: Polyadenosine-diphosphate-ribose polymerase, PI3K: phosphoinositide 3-kinases, siRNA: small interfering RNA.

## Data Availability

Not applicable.

## Supplementary Materials

Link to Prospero application: https://www.crd.york.ac.uk/PROSPERO/view/CRD420251078792 accessed on 23 June 2025.

## Abbreviations

OPN (Osteopontin), CD44 (cluster of differentiation 44), messenger ribonucleic acid (mRNA), PI3K (phosphoinositide 3-kinases), NF-ĸB (nuclear factor kappa-light-chain-enhancer of activated B cells), MAPK (mitogen-activated protein kinase), WNK-1 (lysine-deficient protein kinase 1), PRAS40 (proline-rich AKT substrate 40), FAK (focal adhesion kinase), AKT (protein kinase B), ERK (extracellular signal-regulated kinase), VEGF (vascular endothelial growth factor), COL11A1 (collagen type XI alpha 1), EC (endometrial cancer), DNA (deoxyribonucleic acid), Src (proto-oncogene tyrosine-protein kinase), EMT (Epithelial–Mesenchymal transition), NK (natural killer), ECM (extracellular matrix), OS (overall survival), DFS (disease-free survival), MMPs (Metalloproteinases), FIGO (International Federation of Gynecology and Obstetrics), TNFα (tumor necrosis factor alpha), CA125 (cancer antigen 125), CEA (carcinoembryonic antigen), CA19-9 (carbohydrate antigen 19-9), HE4 (human epididymis protein 4), CA15-3 (cancer antigen 15-3).
